# The Effects of Meaning and Emotional Content of a Sentence on the Kinematics of a Successive Motor Sequence Mimiking the Feeding of a Conspecific

**DOI:** 10.3389/fpsyg.2016.00672

**Published:** 2016-05-09

**Authors:** Elisa De Stefani, Doriana De Marco, Maurizio Gentilucci

**Affiliations:** Department of Neuroscience, University of ParmaParma, Italy

**Keywords:** spoken sentences, emotion, prosody, meaning, human kinematics, voice spectra

## Abstract

**Aim:** Do the emotional content and meaning of sentences affect the kinematics of successive motor sequences?

**Material and Methods:** Participants observed video-clips of an actor pronouncing sentences expressing positive or negative emotions and meanings (related to happiness or anger in Experiment 1 and food admiration or food disgust in Experiment 2). Then, they reached-to-grasp and placed a sugar lump on the actor’s mouth. Participants acted in response to sentences whose content could convey (1) emotion (i.e., face expression and prosody) and meaning, (2) meaning alone, or (3) emotion alone. Within each condition, the kinematic effects of sentences expressing positive and negative emotions were compared. Stimuli (positive for food admiration and negative for food disgust), conveyed either by emotion or meaning affected similarly the kinematics of both grasp and reach.

**Results:** In Experiment 1, the kinematics did not vary between positive and negative sentences either when the content was expressed by both emotion and meaning, or meaning alone. In contrast, in the case of sole emotion, sentences with positive valence made faster the approach of the conspecific. In Experiment 2, the valence of emotions (positive for food admiration and negative for food disgust) affected the kinematics of both grasp and reach, independently of the modality.

**Discussion:** The lack of an effect of meaning in Experiment 1 could be due to the weak relevance of sentence meaning with respect to the motor sequence goal (feeding). Experiment 2 demonstrated that, indeed, this was the case, because when the meaning and the consequent emotion were related to the sequence goal, they affected the kinematics. In contrast, the sole emotion activated approach or avoidance toward the actor according to positive and negative valence. The data suggest a behavioral dissociation between effects of emotion and meaning.

## Introduction

Language is a cognitive function that has the possibility to modify the relations between human listeners/observers (Gentilucci and Dalla Volta, 2008). A tight link has been suggested between language and motor control (Gentilucci and Dalla Volta, 2008; Gentilucci and Campione, 2011; De Stefani et al., 2013), and this is supported by previous evidence (Gentilucci et al., 2001; Gentilucci, 2003). In a recent experiment (De Stefani et al., 2013), participants watched gestures or words, which could be emblems (e.g., “okay”) or requests (e.g., “give me”), with either positive or negative valence, or meaningless signals. At the end of the presentation, they had to perform a reaching-grasping action if the signal was meaningful, but to refrain from moving when the signal was meaningless. The words with negative valence were associated with faster movements, and this effect was greater when the signal was a request.

This study, however, did not dissociate the type of the interaction request from the word meaning. For an example, the word (or gesture) “stop” commands blocking a movement, as the word meaning is tightly associated with the block of the actual action. Conversely, a sentence with a negative mood toward others (expressed by meaning and/or emotional state) might induce in a listener/observer the desire to refrain from interacting with the interlocutor. The type of act toward people consequent to an emotion (e.g., related to happiness or anger and food admiration or food disgust), can implicitly favor or disengage relations with others (Chen and Bargh, 1999). In general, the emotional aspect of the sentence can influence the execution of actions of the listener/observer, as it has been supported for meaning (Chen and Bargh, 1999; Freina et al., 2009). Freina et al. (2009) reported that participants were faster in responding when pressing a far button after visual presentation of positive words and a near button for negative words, as if they simulated reaching for something good and avoiding for something bad. However, they measured the time to response only, rather than the kinematics of the entire action. The kinematics of an entire action gives an insight into both the planning and the control of movement execution, whereas the time to response (RT) gives an insight into the time required to plan a movement. Consequently, the kinematics can provide more information on the effects of valence on behavior. In addition, in their study Freina et al. (2009), presented the stimuli visually rather than acoustically and, consequently, the effect of prosody was not tested.

Other studies (Solarz, 1960; da Gloria et al., 1994; Chen and Bargh, 1999; Rotteveel and Phaf, 2004; Marsh et al., 2005; Rinck and Becker, 2007) found an opposite effect. Pushing a lever, that is refusing an object (or a person), according to the authors, was faster when it was successive to a negative word whereas pulling a lever that is approaching an object was slower when the word was positive. Many theories suggest a close link between emotions and actions, such as, for example, approach and avoidance behaviors (Frijda, 1986). Nevertheless, studies investigating the role of both prosody and face emotional expressions and the semantic content (meaning) on motor actions are remarkably sparse. van Rijn et al., (2005), using repetitive transcranial magnetic stimulation (rTMS), investigated the role of the right frontoparietal operculum in the detection of emotional content conveyed by prosody. The authors showed that, the detection of emotional prosody was significantly impaired after inhibitory stimulation of in right frontal regions, whereas the detection of emotional semantics was not affected, suggesting a possible dissociation between the two processes. In a study using the fMRI technique, Warren et al. (2006) demonstrated that a network including the left human premotor cortex, activated during facial movements, was also active during auditory processing of affective non-verbal vocalizations.

Pitch and intensity of speech are considered as parameters expressing prosody (Pell, 2001; Wildgruber et al., 2006). In the present study, we first aimed at confirming that the positive (happiness) and negative (anger) emotion expressed by a sentence differently influences voice spectra and, secondly, determining whether it affects even the kinematics of a subsequent motor sequence of reaching-grasping and placing. The sequence resembled the feeding of a conspecific. Specifically, we determined whether the negative valence induced an increase or decrease in the velocity of the successive action. Increase could be an index of shortening of the interaction of the participant with an individual to whom the action was directed. Decrease could demonstrate that the negative emotion interfered with the participant’s interaction with the conspecific. Thirdly, we aimed at comparing this difference if present with the one potentially induced by the meaning valence (positive or negative). Finally, we aimed at finding whether the distinction between the processing of emotional prosody and meaning of sentences previously described at anatomical level (van Rijn et al., 2005) exists at a behavioral level, even.

In the present study, we analyzed the kinematics of a feeding motor sequence after a video-clip showed an actress pronouncing aloud sentences whose content could be conveyed by both meaning and emotion (expressed by both prosody and congruent facial expression), or the sole emotion or the sole meaning with either positive or negative valence. A first hypothesis was that, according to a previous study (Freina et al., 2009), positive valence (happiness) could induce faster approach, whereas negative valence (anger) could induce slower movement due to interference. Consequently, we expected a facilitation in response to positive valence, whereas we expected an interference in response to negative valence. We decided to present both acoustic (speech) and visual (facial expression of the actor) stimuli in order to maintain the mimicked interaction (i.e., a communicative request followed by a motor sequence of feeding) as ecological as possible. A second hypothesis was that the meaning effects could be dissociated from the emotion effects. The meaning valence could have an effect that could depend on the conveyed valence in general, but also on the relevance of the meaning with respect to the aim of the sequence, which the participant should execute after presentation of the speaking actor.

## Materials and Methods

### Experiment 1

#### Participants

Thirteen right-handed (Oldfield, 1971), naïve volunteers (eight females and five males, age 21–27) participated in the experiment. All participants were native Italian speakers. The study received approval from the local ethical committee (Comitato Etico per Parma) and was conducted according to the principles expressed in the Declaration of Helsinki. The participants provided written informed consent.

#### Apparatus, Stimuli, and Procedure

**FIGURE 1:**
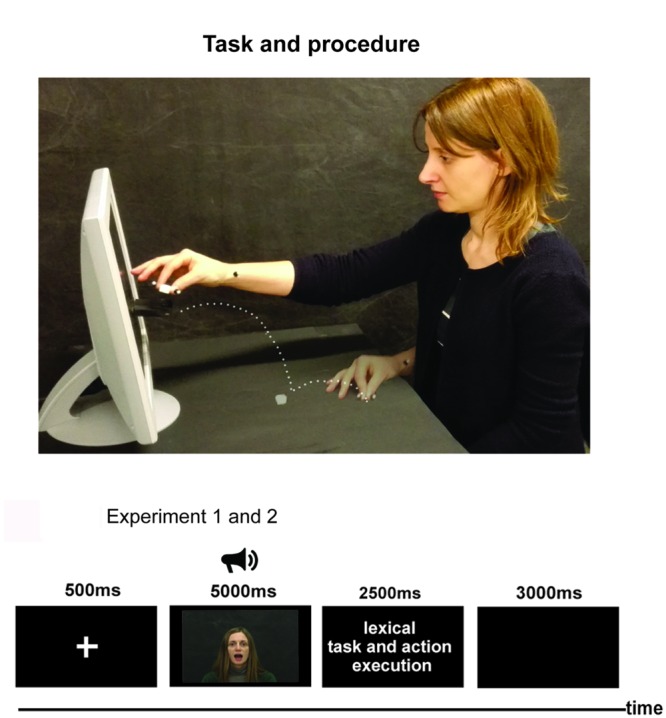
**Task and procedure of Experiments 1 and 2.** The upper part of the figure presents a participant during the execution of reaching-grasping and placing a sugar lump on a container below the mouth of the acress presented by a PC display. Points represent the hand trajectory of the motor sequence. The lower part of the figure represents the procedures of Experiments 1 and 2.

The participant (**Figure 1**) sat comfortably in front of a table, on which he or she placed their right hand with the thumb and index finger in a pinching position (starting position, SP). The SP was along the participant’s mid-sagittal plane and was 20 cm distant from the table edge. The monitor of a PC (19-inch LCD) was placed on the table plane, 70 cm distant from the participant’s forehead. It was set at a spatial resolution of 1024 × 768 pixels and at a temporal resolution of 60 Hz. A sugar lump (1 cm × 1 cm × 1 cm) was positioned on the table plane along the participant’s mid-sagittal plane at a distance of 20 cm from the SP. Other stimuli were presented in the monitor and consisted of video clips during which an actress pronounced aloud a sentence randomly selected out of 10 sentences whose meaning and emotion could be positive (expressing happiness), negative (expressing anger), or neutral (see supplementary material for the entire list of sentences used in Experiments 1 and 2). The following stimuli were presented: sentences with positive meaning and positive emotion (expressed by prosody and face expression), negative meaning and negative emotion, neutral meaning and positive emotion, neutral meaning and negative emotion, positive meaning and neutral emotion, and negative meaning and neutral emotion. We required the actress to assume a facial expression congruent with the prosody. We did not differentiate the effects of the facial expression from those of the prosody. Since both facial expression and prosody are necessary to convey an individual’s emotional status, we thought that the facial expression should be congruent with the prosody, and that both prosody and facial expression should be not emphasized.

Stimuli were chosen on the basis of the results of a task carried out on a separate sample of 30 volunteers. The task was to judge the valence of sentences presented by videos, assigning a score from 1 to 6 according to the emotion valence (score 1–2 negative, 3–4 neutral, 5–6 positive). Since the task aimed at choosing the sentences to present to the participants, we required to judge the overall sentence valence considering together meaning, prosody and facial expression. The sentences with the lowest and highest scores were chosen as negative and positive stimuli, respectively. The mean scores of negative, neutral, and positive sentences were 1.85, 3.31, and 4.98. In an ANOVA, they significantly differed from each other [*F*(2,18) = 71.43, *p* < 0.00001, *post hoc p* < 0.0001]. After pronouncing the sentence, the actress in the video opened her mouth and remained still until the end of trial. The participants performed a go/no-go task. Once the actress’ mouth was opened and when the sentence was meaningful, they had to reach the sugar lump as soon as possible, to pick it up and, finally, to place it on a small container attached to the video display, below the actress’s mouth (**Figure 1**). The sequence resembled the feeding of a conspecific for the following features: the presence of a conspecific giving a piece of food, the opened mouth of the actress, and the chunks of the sequence (grasping a piece of food and placing it on the actress’ mouth). Three blocks of trials were run, in which the following conditions were presented: the first block (meaning plus emotion condition), positive meaning and positive emotion, and negative meaning and negative emotion; the second block, (sole emotion condition) neutral meaning and positive emotion, and neutral meaning and negative emotion; the third block (sole meaning condition) positive meaning and neutral emotion, and negative meaning and neutral emotion. Another 10 trials with meaningless sentences (see **Data Sheet 1**) were randomly added to each block. In total, each block consisted of 30 trials (10 positive, 10 negative meaningful and 10 meaningless trials). Within each block, the stimuli were randomly presented. The order of block presentation was counterbalanced across subjects. In total, 90 trials were run by each participant.

#### Data Recording

The movements of the participants’ right arms were recorded using the 3D-optoelectronic SMART system (BTS Bioengineering, Milano, Italy). This system consists of six video cameras detecting infrared reflecting markers (5-mm-diameter spheres) at a sampling rate of 120 Hz. The spatial resolution was 0.3 mm. We attached two reflective markers to the nails of the participants’ right thumb and index finger, in order to analyze the grasp kinematics, by recording the time course of the distance between the thumb and the index finger. The time course of the grasp is constituted by an initial phase of the fingers opening up to a maximum (maximal finger aperture), followed by a phase of the finger closing on the object (Jeannerod, 1988). Another marker was attached to the wrist and was used to analyze the kinematics of reach and place. The data from the recorded movements were analyzed using homemade functions developed using MATLAB (R2008b). A Gaussian low pass smoothing filter (sigma value: 0.93) was applied to the recorded data. The time course of reach-grasp and lift was visually inspected in order to identify the beginning and the end of the entire movement; the beginning of grasp, reach and lift phases was inspected with different criteria: the beginning of the grasp was considered to be the first frame in which the distance between the two markers placed on the right finger tips increased with respect to the previous frame; the end of the grasp was the first frame after the beginning of finger closing in which the distance between the two right fingers did not change with respect to the previous frame; and the beginning of the reaching, corresponding to the start of the movement, was chosen as the first frame during which the displacement of the reaching marker along any Cartesian body axis increased with respect to the previous frame. To determine the end of the reach we calculated separately for each of the *X, Y*, and *Z* axes the first frame following movement onset in which the *X, Y*, and *Z* displacements of the reaching marker did not change compared to the previous frame. Then, the frame endpoint temporally closer to the grasping end frame was chosen as the end of the reach. The frame immediately successive to the reach end was considered the lift beginning, while the lift end corresponded to the frame in which the highest point of the hand path was reached during placement. We measured the following grasp parameters: finger opening peak velocity and peak acceleration. We also analyzed the reach and place peak velocity and reach peak acceleration. We computed these velocity and acceleration peaks for reaching and grasping because we were interested on how meaning and emotion affected the interactions of the participant with the object (the grasp) and the conspecific (the reach). Two loudspeakers (Creative, Inspire T10) connected to the PC were used to present the acoustic stimuli in the experiments. In a separate session, we recorded the voice of the actress in 10 repetitions of each sentence by means of a light-weight dynamic headset microphone. The frequency response of the microphone was 50–15,000 Hz. The microphone was connected to a PC by a sound card (16 PCI Sound Blaster; Creative Technology Ltd., Singapore), and audio was acquired using the Avisoft SAS Lab professional software (Avisoft Bioacoustics, Germany). The actress’ voice parameters (pitch_ that is the variation in the fundamental frequency calculated for each interval of acquisition_ and intensity, both averaged over the entire sentence) were successively measured using the PRAAT software^1^ set as follows. Pitch: range (Hz): 75–500, analysis method: autocorrelation, intensity range (dB): 50–100, average method: mean energy, silent threshold: 0.03, voicing threshold: 0.45. This setting allowed to exclude the silent periods from the analysis.

#### Data Analysis

Repeated measures ANOVAs were carried out on the voice spectra parameters of the actress’ voice and on the mean values the reaching-grasping, and placing parameters of the participants The within- subject factors were sentence valence (positive – happiness versus negative – anger) and sentence modality (meaning plus emotion, that is prosody and face expression, versus sole meaning, versus sole emotion). In all analyses, *post hoc* comparisons were performed using the Newman–Keuls procedure. The significance level was fixed at *p* = 0.05. Sphericity of the data was verified before performing statistical analysis (Mauchly’s test, *p* > 0.05). In Experiment 1 all variables were normally distributed as verified by Kolmogorov–Smirnov Test (*p* > 0.05). $\eta_{p}^{2}$ was also calculated.

#### Pitch, Intensity, and Duration of the Sentences Pronounced by the Actress

The interaction between valence and modality significantly affected pitch [*F*(2,16) = 3.9, *p* = 0.05, $\eta_{p}^{2}$ = 0.3, **Figure 3** and intensity [*F*(2,16) = 21.3, *p* = 0.00003, $\eta_{p}^{2}$ = 0.7, **Figure 3**]. *Post hoc* comparisons showed that the negative emotion induced an increase in the two parameters compared to the positive emotion (*p* = 0.02, *p* = 0.0001). The same thing occurred when the emotion was associated with the congruent meaning (*p* = 0.02, *p* = 0.0001). In this condition, however, pitch and intensity were lower than in the sole emotion condition (*p* = 0.02, *p* = 0.01). No significant difference was found between positive and negative valence in the condition of sole meaning for pitch and intensity (*p* = 0.48, *p* = 0.21). Positive and negative meaning induced a decrease in pitch and intensity, compared to the remaining conditions (*p* < 0.02, *p* < 0.0001). Sentence duration was affected by factor modality [*F*(2,18) = 20.41, *p* = 0.0001, $\eta_{p}^{2}$ = 0.7, meaning 1.65 s, meaning plus emotion 2.03 s, emotion 2.46 s, all comparisons were significant, *p* = 0.008] but not by factor valence [*F*(1.9) = 0.7, *p* = 0.8]. The modality per valence interaction was not significant for sentence duration [*F*(2,18) = 1.1, *p* = 0.35].

### Experiment 2

#### Participants

A new sample of fourteen female, right-handed (Oldfield, 1971) and naïve volunteers (age 21–26) participated in the experiment. All participants were native Italian speakers. They provided written informed consent to participate in the study, which was approved by the local ethical committee (Comitato Etico per Parma) and was conducted according to the principles expressed in the Declaration of Helsinki. Since the participants were presented either with face and voice expressing emotions related either to food admiration or disgust, they were assessed with EDI-3 (Eating Disorders Inventory-3; Garner, 2008, Giunti O.S.) in order to exclude any symptom related to anorexia or bulimia. Consequently, we tested whether the variation in sequence kinematics could be ascribed to behaviors related to these eating disorders, that result more common in young female individuals (Smink et al., 2012). Note that effects of anorexia or bulimia were possible only in Experiment 2 because the expressed emotions were related to food admiration or food disgust rather happiness or anger as in Experiment 1.

#### Apparatus, Stimuli, and Procedure

The apparatus was the same as in Experiment 1 (**Figure 1**). The stimuli presented in the monitor consisted of video-clips during which the same actress as in Experiment 1 pronounced aloud 1 out of 10 sentences selected at random, whose meaning and emotion could express food admiration or food disgust, or could be neutral (see **Data Sheet 1** for the entire list of sentences used in Experiments 1 and 2). The sentences were divided into three blocks: the block 1, meaning plus emotion (sentences whose meaning, prosody and face expression were related to food admiration/disgust) the block 2, sole emotion (sentences pronounced with a prosody and face expression related food admiration/disgust, but with a neutral meaning) and the block 3, sole meaning (sentences with meaning related to food admiration/disgust but pronounced with a neutral prosody, and face expression). The actress’ facial expression was congruent with the emotion related to food admiration or disgust. The stimuli were chosen according to the results of a task in which a different sample of 30 volunteers participated. The task was to judge the valence of the videos assigning a score from 1 to 6 to food admiration/disgust expressed by the sentence (score 1–2, food disgust, 3–4 neutral, 5–6 food admiration). The mean scores of negative, neutral, and positive sentences were 1.42, 3.31, and 5.08. In an ANOVA, they significantly differed from each other [*F*(2,18) = 369.9 *p* < 0.00001, *post hoc p* = 0.0002]. The remaining procedure was the same as in Experiment 1. In total, each block consisted of 30 trials (10 positive, 10 negative meaningful, and 10 meaningless trials). The order of block presentation was counterbalanced across subjects. In total, each participant was presented with 90 trials.

#### Data Recording

Data recording was the same as in Experiment 1. The movements of the participants’ right arm were recorded using the 3D-optoelectronic SMART system (BTS Bioengineering, Milano, Italy). We measured the following parameters: peak velocity of finger opening and closing, reach, and place peak velocity and reach peak acceleration. In a separate session, we recorded the voice of the actress pronouncing aloud the sentences in each condition by means of a light-weight dynamic headset microphone, as in Experiment 1. The microphone was connected to a PC by a sound card and audio was acquired using the Avisoft SAS Lab professional software (Avisoft Bioacoustics, Germany). The actress voice parameters (pitch, intensity, and sentence duration) were successively measured and analyzed as in Experiment 1, using the PRAAT software^2^.

#### Data Analysis

Repeated measures ANOVAs were carried out on the mean values of the voice parameters of the actress and the reaching-grasping, and placing parameters of the participants. The within-subject factors were sentence valence (food admiration _ positive versus food disgust_ negative) and sentence modality [meaning and emotion (prosody and face expression), versus sole meaning, versus sole emotion]. The significance level was fixed at *p* = 0.05. In all analyses, *post hoc* comparisons were performed using the Newman–Keuls procedure. The assumption of sphericity was assessed using Mauchly test. If the sphericity assumption was violated (significant results in Mauchly test), the degrees of freedom were corrected using the Greenhouse Geisser estimates of sphericity. All variables were normally distributed as verified by Kolmogorov–Smirnov Test (*p* > 0.05). $\eta_{p}^{2}$ was also calculated.

#### Pitch, Intensity, and Duration of the Sentences Pronounced by the Actress

**FIGURE 2:**
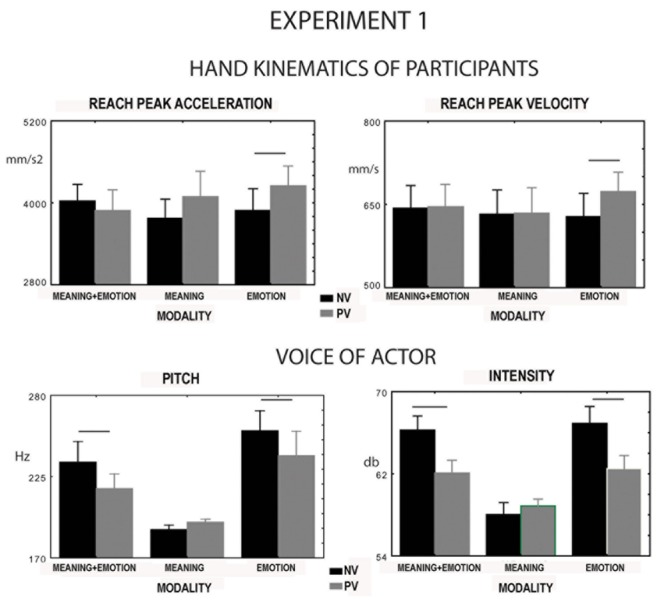
**Experiment 1.** Parameters of hand kinematics of the participants and voice spectra of sentences presented to a conspecific by a video. Upper row: variation in reach peak acceleration and velocity of participants during reaching-grasping and placing a sugar lump on the conspecific’s mouth. The sentence features could be meaning and emotion, meaning only, or emotion only, whereas the sentence valence could be either positive or negative. Lower row: variation in pitch and intensity of the voice of the actress pronouncing the sentences when feature and valence varied. PV, positive valence; NV, negative valence. Significances between positive and negative valence for each feature are only reported.

The factors modality and valence affected pitch [modality: *F*(2,18) = 15.71, *p* = 0.00011, $\eta_{p}^{2}$ = 0.63, meaning plus emotion, 217.5 Hz, emotion, 209.0 Hz, meaning, 202.9 Hz; in *post hoc* all comparisons were significant, *p* < 0.03, valence [*F*(1,9) = 20.2, *p* = 0.0015, food disgust, 215.1 Hz, food admiration, 204.6 Hz]. Factor modality affected intensity [*F*(1.153,18) = 12.0, *p* = 0.005, $\eta_{p}^{2}$ = 0.57, meaning plus emotion, 73.0 db, emotion, 70.3 db, meaning, 72.5 db, *post hoc* comparisons: emotion versus meaning and meaning plus emotion, *p* < 0.0018]. Mauchly’s test indicated that the assumption of sphericity had been violated [*X*(2) = 10.6, *p* = 0.005]; consequently, the degrees of freedom were adjusted using 𝜀-Geisser correction (𝜀 = 0.58). Factor valence also affected intensity [*F*(1,9) = 53.4, *p* = 0.00005, food disgust, 73.2 db, food admiration, 70.6 db]. The interaction between modality and valence was significant [*F*(2,18) = 10.95, *p* = 0.00078, $\eta_{p}^{2}$ = 0.55, **Figure 2**]. In modality emotion, a decrease in intensity was found for food admiration compared to food disgust (*p* = 0.00019). The same occurred for the modality meaning (*p* = 0.0007, **Figure 2**). The food admiration in the modality emotion induced a significant decrease in this parameter as compared to the other conditions (*p* < 0.01, **Figure 2**). Factor modality affected sentence duration [*F*(1.096,18) = 58.2, *p* = 0.0001, $\eta_{p}^{2}$ = 0.87, meaning plus emotion, 1.99s, emotion, 1.80s, meaning, 2.49s, all comparisons were significant, *p* < 0.0009]. Mauchly’s test indicated that the assumption of sphericity had been violated [*X*(2) = 13.9, *p* = 0.0001]; consequently, the degrees of freedom were adjusted using Greenhouse-Geisser correction (𝜀 = 0.55). After correction, factor valence affected sentence duration [*F*(1,9) = 4.82, *p* = 0.05, $\eta_{p}^{2}$ = 0.34, food disgust, 1.98s, food admiration, 2.21s]. The interaction between modality and valence was not significant [*F*(1.152,18) = 3.6, *p* = 0.082, $\eta_{p}^{2}$ = 0.29]. **Figure 2** shows the significance in the comparison between food admiration and food disgust in the three modalities. Only in modality meaning plus emotion the sentence duration significantly increased in the case of food admiration (*p* = 0.001). Other significances not presented in **Figure 2** are the following: concerning food admiration and food disgust, the comparisons between emotion and meaning (*p* = 0.0002) and between emotion and meaning plus prosody (*p* = 0.0003) were significant.

## Results

### Experiment 1

We have reported the percentage of errors per condition in **Table 1**. The total percentage of errors was 2.18%, corresponding to an accuracy of 97.82%. The number of errors were few and constant (**Table 1**); consequently, we cannot explain the kinematic variation as result of variation in task accuracy.

**Table 1:** Errors (percentage with respect to total number of trials in Experiments 1 and 2).

Condition	Experiment 1	Condition	Experiment 2
Prosody – positive valence	2.30	Prosody – positive valence	2.4
Prosody – negative valence	1.53	Prosody – negative valence	3
Meaning – positive valence	1.53	Meaning – positive valence	6
Meaning – negative valence	2.30	Meaning – negative valence	4.2
Prosody + Meaning – positive valence	0.76	Prosody + Meaning – positive valence	5.4
Prosody + Meaning – negative valence	4.61	Prosody + Meaning – negative valence	1.8

#### Reaching-Grasping and Placing Kinematics of the Participants

**FIGURE 3:**
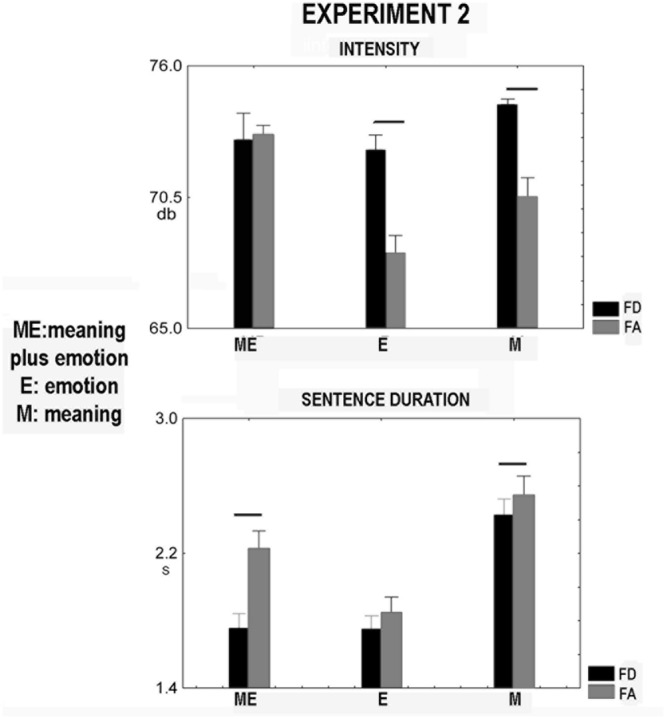
**Experiment 2.** Significant voice spectra parameters of sentences pronounced aloud by the actress. They were the following: intensity and sentence duration. FD, food disgust; FA, food admiration. Whiskers are SE, horizontal bars indicate statistical significance.

In the ANOVAs no main effect nor interaction [e.g., interaction between valence and modality; *F*(2,24) = 2.9, *p* = 0.073, *F*(2,24) = 2.4, *p* = 0.112] significantly modulated finger opening peak velocity and peak acceleration. In contrast, the interaction between valence and modality did affect reach peak velocity [*F*(2,24) = 3.5, *p* = 0.05, $\eta_{p}^{2}$ = 0.22, **Figure 3**] and reach peak acceleration [*F*(2,24) = 3.4, *p* = 0.031, $\eta_{p}^{2}$ = 0.25, **Figure 3**]. *Post hoc* tests showed a significant variation in the two parameters when presenting sentences with sole emotion varying in valence. Specifically, a significant increase in velocity was observed when the valence was positive (*p* = 0.02, *p* = 0.04, **Figure 3**). In contrast, no significant effect of valence was found for the sole meaning (*p* = 0.8, *p* = 0.1) and meaning plus emotion (*p* = 0.8, *p* = 0.5) conditions. No effect on place peak velocity for any of the investigated factors was observed.

### Experiment 2

**Table 1** shows the percentage of errors per condition. The total percentage of errors was 3.8%, corresponding to an accuracy of 96.20%.

#### Reach-Grasp and Place Kinematics of the Participants

**FIGURE 4:**
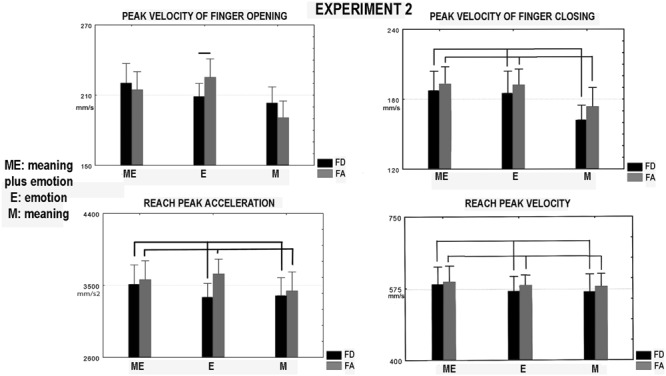
**Experiment 2.** Parameters of hand kinematics of the participants during performing the feeding sequence. They were the following: peak velocity of finger opening and closing, reach peak velocity, and acceleration. Other conventions as in **Figures 2** and **3**.

In the ANOVA the interaction between valence and modality [*F*(2,26) = 4.46, *p* = 0.019, $\eta_{p}^{2}$ = 0.73, **Figure 4**], affected finger opening peak velocity this parameter related to food admiration was greater in the emotion condition than that related to food disgust (*p* = 0.06). Factor valence affected finger closing peak velocity which was greater in the food admiration than food disgust condition [*F*(1,13) = 10.7, *p* = 0.006, $\eta_{p}^{2}$ = 0.86, **Figure 4**]. Factor valence influenced reach peak acceleration [*F*(1,13) = 7.84, *p* = 0.015, $\eta_{p}^{2}$ = 0.73, **Figure 4**] and showed a trend to significance for reach peak velocity [*F*(1,13) = 3.95, *p* = 0.07, **Figure 4**, $\eta_{p}^{2}$ = 0.45]. Both parameters increased in the condition food admiration compared to food disgust. Note that, in the condition of sole meaning of Experiment 2 (emotion was neutral) valence affected finger closing peak velocity in association with the other modalities. Neither factor nor interaction between factors affected place peak velocity.

## Discussion

In the reported experiments, we investigated whether the valence of an emotional status (expressed by prosody plus facial expression) and/or the meaning of a sentence automatically affected the kinematics of a successive motor sequence mimicking the feeding of a conspecific. Our first hypothesis was that, according to the results of a previous study (Freina et al., 2009) positive valence would have induced facilitation and, consequently, faster approach of the participant’s hand toward the speaker’s mouth. On the other side, we expected a slowing down of the approaching movement in response to negative valence, due to avoidance and/or interference. The present results confirmed the data by Freina et al. (2009). However, as reported in Introduction, other studies (Solarz, 1960; da Gloria et al., 1994; Chen and Bargh, 1999; Rotteveel and Phaf, 2004; Marsh et al., 2005; Rinck and Becker, 2007) found that hand velocity varied with valence but increasing instead of decreasing when the vocal message was negative. We can explain this contrasting datum considering that in most of those studies the participant was required to either push or pull a lever in response to a word/sentence. The response to a negative valence stimulus could be quickly pushing a lever against the object/person related to the action. Conversely, the response to a positive valence could be pulling slowly a lever in order to approach carefully the self. This is in accordance with the fact pushing could be interpreted as rejecting (refusing) a negative stimulus, whereas pulling could be interpreted as taking possession of a positive stimulus. Refusing could increase push velocity whereas taking could decrease velocity in order to increase movement accuracy. In contrast, in the present study, a sequence was executed with an aim, i.e., feeding. Feeding was faster when the sentence was positive, that is related to food admiration because it could facilitate the hand approach of the target (reaching-grasping the sugar lump) before introducing it into the actress’ mouth. The opposite (i.e., an interference) could occur when the word valence was negative. Probably, the type of response, that is moving a lever to respond, influenced the kinematics planning differently from planning a feeding sequence.

Rusconi et al. (2005) proposed that variation in pitch could change localization of the stimulus toward which the action was directed. That is, higher pitch for negative words induced an illusion of increasing target height, and consequently velocity. The opposite occurred for positive words. In other words, target localization and consequent kinematics might be affected by pitch. However, (see **Figure 3**) an effect on reach peak velocity and acceleration was not found in the modality meaning plus prosody even if pitch varied with valence. Consequently, an effect due to pitch variation cannot be proposed.

The second hypothesis was that the meaning effects could be dissociated from those of valence. The effects on the sequence kinematics, that we found in the present study could be ascribed to the voice spectra of the sentences pronounced aloud by the actress. The meaning and valence could be related to possible interactions with conspecifics, even if the referent was neither a single person nor a group of persons. Valence expressed by the meaning, as well as emotion, could be positive (expressing happiness) or negative (expressing anger, Experiment 1). When only the meaning valence varied or when it was associated with the congruent emotion, no effect of valence was observed on hand kinematics. This result excludes the possibility that the task requiring to act if the sentence was meaningful was responsible for variation in hand kinematics. Indeed, even if the sentence was meaningful, its valence did not affect movement. Conversely, a possible explanation is that the sentence meaning was unrelated to the sequence goal; that is, the feeding of a conspecific. Since emotions evoked by the sentences were not strictly relevant for the feeding sequence as food admiration and food disgust are (see Ferri et al., 2010), they did not influence the sequence. In order to prove this hypothesis, we have conducted Experiment 2, in which the emotions expressed food admiration or food disgust. We found that these emotions related to feeding did induce an effect even in the modality meaning. We concluded that, in Experiment 1, the meaning of the sentence was understood (as the task required), but it was irrelevant for the execution of the sequence. Consequently, the possibility of affecting the sequence decayed. In contrast, the effects of meaning emerged only when it was specifically relevant to the final aim of the sequence (Experiment 2). In contrast with the lack of any effect in the case of meaning unrelated to the sequence, an effect of valence was observed when only the emotion valence varied. That is, it occurred when the meaning was neutral and emotion could convey either a positive or a negative emotional state. Neutral meaning did not establish a degree of relevance of the sequence aim (i.e., the relevance remained undetermined). Consequently, in this case, the valence only could be effective. **Figure 3** shows that, in Experiment 1, the kinematic effects of the valence were mainly provided by the positive prosody. In fact, the kinematic effects of the negative valence of the sole prosody did not differ from the other conditions of negative valence. In contrast, the positive valence of the sole emotion induced a variation in the kinematic parameters in comparison with the other conditions of positive valence. This did not occur in Experiment 2, where in all modalities the positive valence was responsible for kinematic changes compared to negative valence. This is in line with the results by Seidel et al. (2010), who found approach tendency with happiness and avoidance tendency with anger for faces expressing the Ekman and Friesen’s (1981) emotions. In sum, the valence can be effective for emotions relevant to the aim of the sequence (Ekman and Friesen, 1981). However, its effect may be independent of the meaning when we consider the sole emotion. Here, we distinguish only between approach and avoidance effects, independently of the actions to execute and the specific meaning of the emotional state (Gentilucci et al., 2012). An approach tendency produces quicker movements directed toward a conspecific, whereas avoidance interferes with the approach, slowing down movement (van Dantzig et al., 2008). The results of a previous study (De Stefani et al., 2013) seem to be in contrast with the present data. In fact, in that study, faster movements were observed in the case of negative valence, whereas in the present one faster movements were observed in the case of positive valence, according to previous studies (Solarz, 1960; da Gloria et al., 1994; Chen and Bargh, 1999; Rotteveel and Phaf, 2004; Marsh et al., 2005; Rinck and Becker, 2007). This discrepancy is probably explainable by the interaction with the receiver required by the task of the present study (a sequence of feeding), which was not required in the previous study where the reaching-grasping phase was not concatenated with a place, resulting in this case, in an action not directed toward the receiver.

Thus, the valence effects did not depend on the relationship with the actor, but on an automatic response to the stimulus independent of the required movement (De Stefani et al., 2013). Summing up, the weak relevance of the expressed emotional state for the action accomplishment resulted in the absence of meaning effects on the motor sequence kinematics. However, even in this situation, an effect of valence producing approach and avoidance according to prosody was observed. This different effect of the emotion and meaning on hand kinematics shows a clear dissociation between the two sentence modalities. This emerged when the relevance of the meaning for the sequence aim was weak. This dissociation decayed when sentences’ meanings were highly related to the motor sequence. The analysis of the speech parameters confirmed that pitch and intensity depended on emotion rather than meaning. Thus, in the case of sole emotion, the reported hand kinematic modulations might also be due to listening/observing and automatically simulating the emotion. The simulation commands might be transferred from the mouth control system, to the hand control system modifying hand kinematics. This in line with a hypothesis suggesting the existence of a system controlling both mouth and hand movements (Gentilucci and Campione, 2011; Gentilucci et al., 2012). The emotion effects were stronger on reach rather than grasp parameters since it establishes how to interact with another individual (e.g., the receiver of the action sequence), rather than how to interact with the object of the sequence (e.g., the sugar lump). This evidence further confirms that meaning and prosody were dissociated at behavioral level, in line with the dissociation reported at a neuroanatomical level (van Rijn et al., 2005). Prosody acted on reaching, whereas meaning did not.

Pihan (2006) measured direct current (DC) EEG potentials during presentation of pairs of declarative sentences with either happy, sad, or neutral intonation, which were different in pitch and duration. Results demonstrated a predominant role of the right hemisphere in processing emotions from the tone of voice, irrespective of emotional valence. However, right hemisphere involvement is modulated by diverse speech and language-related conditions that were associated with a left hemisphere participation in valence processing.

Which are the relations between prosody and motor control at neuroanatomical level? Data obtained from patients with primary focal dystonia evidenced a significant impairment in auditory prosody recognition compared to an healthy population (Nikolova et al., 2011).

Primary dystonia is a movement disorder attributed mainly to basal ganglia dysfunction. Besides motor control, striatopallidal structures are known to implement also non-motor functions including processing of cognitive and emotional information (Schröder et al., 2010). In sum, both right and left cortices are involved in prosody processing and subcortical structure like Basal Ganglia in integration of prosody with movement control. Has the prosody a social function, as it can express a request to an individual? Our idea is that, since prosody translates an emotional state into an interactive behavior with the listener, it does have a social function. Moreover, the fact that prosody affects reach suggests a modification of the interaction with other individuals and, thus, it is implicated in social behavior.

## Author Contributions

All authors listed, have made substantial, direct and intellectual contribution to the work, and approved it for publication.

## Conflict of Interest Statement

The authors declare that the research was conducted in the absence of any commercial or financial relationships that could be construed as a potential conflict of interest.

## Footnotes

1 www.praat.org

2 www.praat.org

## Supplementary Material

DATA SHEET 1 **List of the positive, negative, neutral and meaningless sentences presented to the participants in Experiments 1 and 2.** In the line below, translation in English is reported.
