# Supplementary material for: The Effects of Meaning and Emotional Content of a Sentence on the Kinematics of a Successive Motor Sequence Mimiking the Feeding of a Conspecific
Source: Front Psychol. 2016 May 9;7:672. doi: 10.3389/fpsyg.2016.00672 (PMC4860854; doi:10.3389/fpsyg.2016.00672)
Supplement: DATA SHEET 1 — List of the positive, negative, neutral and meaningless sentences presented to the participants in Experiments 1 and 2. In the line below, translation in English is reported. [file Data_Sheet_1.DOC]

**Stimuli (sentences of experiments 1 and 2)**

**Positive Sentences (Happiness)**

Sono felice in mezzo agli altri

I am happy when I am in company with other people.

Mi piace fare nuove conoscenze

I like to meet new people.

Ho fiducia nelle persone

I have faith in people.

Sono sempre disponibile con le persone

I am always at someone’s disposal.

Collaborare con gli altri mi stimola

Cooperating with others stimulates me

Ascolto con piacere i consigli degli altri

I listen to the advice of others with pleasure.

Mi piace chiacchierare

I like to chat.

Mi piace stare in compagnia

I enjoy being in the company of others.

Mi piace aver vicino gli altri

I enjoy being close to others

Oggi vorrei festeggiare con gli amici

To-day I would like to celebrate with my friends.

**Negative Sentences (Angry experiment 1)**

Spaccherei la faccia a chi mi sta intorno

I would punch people nearby me.

Non voglio essere disturbato

I do not want to be disturbed.

Trattare con gli altri mi mette di pessimo umore

Dealing with others puts me in a bad mood.

La presenza di altre persone mi infastidisce

The presence of others annoys me.

Non ho tempo per nessuno

I have no time for anyone.

Nessuno mi interessa

I am not interested in anyone

Sono proprio rabbioso

I am really furious.

Spesso ho scoppi d’ira col prossimo

Frequently, I have outbursts of anger towards neighbors.

Voglio star solo

I want to be alone.

Ce l’ho con tutti

I am angry with everyone.

**Neutral Sentences (experiments 1 and 2)**

La camera ha due finestre

There are two windows in the room

Il computer è acceso

The computer is on.

L’armadio è antico

The closet is antique.

La sedia è rotta

The chair is broken.

La macchina è sotto casa

My car is parked close to my home.

La porta è aperta

The door is open.

In un anno ci sono dodici mesi

There are twelve months in one year.

Il riscaldamento è spento

The heating is off.

La frutta è matura

The fruit is ripe.

La lampadina è fulminata

The light bulb is blown.

**Meaningless Sentences (experiments 1 and 2)**

La faccia mi spacca intorno

the face splits around

Il tempo non è cotto per nessuno

Time is not cooked for anyone

Proprio interessa a rabbioso

This interests to angry

Gli scoppi hanno spesso la prossima ira

Outbreaks have often the next fury

Veglia sta solo

Wakefulness is only

La fiducia è alle mucche

Trust is in cows

Dispongo sempre le pressioni

I always place pressures

Lo stimolo collabora con patate

The stimulus collaborates with potatoes

Mi chiacchierano in ascolto

I chat in listening

Il festeggiare vendemmia gli amici

Celebrating harvests the friends

| **Positive Sentences (food admiration, experiment 2)** |
| --- |
| Questo cibo è preparato con molta cura  This food is prepared with great care |
| Questo cibo è salutare  This food is healthy |
| Questo cibo ha un ottimo odore |
| This food has an excellent smell  Questo cibo è da manuale |
| This food is cooked as in a recipe  Questo cibo è cucinato benissimo |
| This food is well cooked  Questo cibo mi fa venire l'acquolina in bocca |
| This food makes me drool  Questa pietanza è veramente gustosa |
| This dish is very tasty  Questa pietanza è buonissima |
| This dish is delicious  Questo cibo è molto squisito |
| This food is very palatable  Questa pietanza è la fine del mondo  This dish is “the end of the world” |

|  | **Negative Sentences (Disgust, Experiment 2)** |  |
| --- | --- | --- |
|  | Questa pietanza ha un gusto acido |  |
|  | This dish has an acid taste |  |
|  | Questo cibo ha un pessimo sapore |  |
|  | This food has a bad taste |  |
|  | Questo cibo puzza |  |
|  | This food stinks |  |
|  | Questo cibo è veramente una porcheria  This food is really rubbish |  |
|  | Questo cibo mi disgusta  This food disgusts me |  |
|  | Questa pietanza è stomachevole  This dish is nauseating |  |
|  | Questo cibo mi ripugna  This food repulses me |  |
|  | Questa pietanza mi da il voltastomaco  This dish give me a sensation of disgust |  |
|  | Questo cibo è proprio marcio  This food is just rotten |  |
|  | Questa pietanza è immangiabile |  |

This dish is inedible
